# Predictive factors affecting cecal intubation failure in colonoscopy trainees

**DOI:** 10.1186/1472-6920-13-5

**Published:** 2013-01-19

**Authors:** Hong-Jun Park, Jin-Heon Hong, Hyun-Soo Kim, Bo-Ra Kim, So-Yeon Park, Ki-Won Jo, Jae-Woo Kim

**Affiliations:** 1 Department of Internal Medicine, Yonsei University Wonju College of Medicine, Wonju, Republic of Korea; 2 Division of Gastroenterology & Hepatology, Department of Internal Medicine and Institute of Lifelong Health, Yonsei University Wonju College of Medicine, 162, Ilsan-dong, Wonju, Gangwon-do 220-701, Korea

## Abstract

**Background:**

Successful cecal intubation (SCI) is not only a quality indicator but also an important marker in a colonoscopy trainee’s progress. We conducted this study to determine factors predicting SCI in colonoscopy trainees, and to compare these factors before and after trainees achieve technical competence.

**Methods:**

Design of this study was a cross-sectional studies of two time series design for one year at a single center. From March 2011 to February 2012, a total 2,050 subjects who underwent colonoscopy by four first-year gastrointestinal fellows were enrolled at Christian hospital, Wonju, Republic of Korea. Four gastrointestinal fellows have filled out the colonoscopic documentation. Main outcome measurement was predictive factors affecting cecal intubation failure and learning curves.

**Results:**

Colonoscopy was successfully completed to the cecum in 1,720 patients (83.9%). Success rates gradually increased as trainees performed more colonoscopies: the rate of SCI was 62% in the first 50 cases, and grew to 93% by the 250th case. Logistic regression analysis of factors affecting cecal intubation failure showed that female gender, low BMI (BMI < 18.5 kg/m2), poor bowel preparation, and past history of stomach surgery were more often associated with cecal intubation failure, particularly before the trainees achieved technical competence.

**Conclusion:**

Several patient characteristics were identified that may predict difficulty of cecal intubation in colonoscopy trainees. Particularly, low BMI, inadequate bowel cleansing, and previous stomach operation were predictors of cecal intubation failure before the trainees have reached technical competency. The results could be informative so that trainees enhance the success rate regarding better colonoscopy training programs.

## Background

Colonoscopy is a widely used procedure for the screening and surveillance of colorectal cancer. Colonoscopy and polypectomy have effectively reduced the incidence of colorectal cancer [1,2]. However, providing a safe and optimal colonoscopy is not easy even after considerable training and experience. Successful cecal intubation (SCI) is a primary quality indicator in colonoscopies. SCI is also an important marker in a colonoscopy trainee’s progress. However, cecal intubation rates in the early learning phase have been reported as only 56-75% [3-6]. Furthermore, delayed or failed cecal intubation can cause unfavorable events, such as patient discomfort, barotrauma and consecutive cecal re-insertion failure, especially if being performed by a trainee. Previous studies have reported the various patient-related factors associated with colonoscopy outcomes. These factors included the age, gender, high or low BMI, bowel preparation, and a history of abdominal surgery and/or peritonitis [7-10]. However, there are limited data about how these factors affect cecal intubation in colonoscopy trainees [4,5]. The goal of this study was to determine the factors that affect the failure of cecal intubation and to compare these factors before and after trainees achieve competence.

## Methods

From March 2011 to February 2012, a single-center cross-sectional observational study was conducted at Wonju Christian hospital for 1 year. Colonoscopic indications included screening/surveillance, evaluation of symptoms such as abdominal pain, discomfort, melena. A total of 2,050 colonoscopies were done by four first-year gastrointestinal (GI) fellows. Patients were excluded for the following reasons: (1) urgent colonoscopy, (2) intended therapeutic colonoscopy, (3) colon obstruction, (4) history of colorectal surgery, and (5) patient refusal. Informed consent was obtained before the procedure. The Ethical Committee at Wonju Christian Hospital approved this study protocol.

### Participants

The participants included four first-year GI fellows involved in colonoscopy training for 12 months. None of the participating fellows had any colonoscopy experience before beginning their fellowship. Each of the four GI fellows completed 450–600 colonoscopies throughout the course of 1 year. Oral consent for their participation in the study was obtained.

### Colonoscopy

All colonoscopies ware performed with an Olympus CF-260 video colonoscope (Olympus Optical Co, Ltd, Tokyo, Japan) after preparatory bowel cleansing with a 4-L of polyethylene glycol solution. We used the following bowel preparation scale: excellent (no or minimal solid stool and only small amount of clear fluid), good (no or minimal solid stool with large amount of clear fluid), fair (collections of semisolid debris that are cleared with difficulty) and poor (solid or semisolid debris that cannot be effectively cleared) [11]. Midazolam and propofol were given to the patient on demand as a bolus. 50 mg of IV pethidine was given prior to the procedure, unless contraindicated for that particular patient. We have both a training protocol for colonoscopy trainees based on quality measurement for colonoscopy and a skill assessment form [12]. Initially, the trainees were instructed to understand indications, processes of procedures, medication of sedation, and descriptions of findings followed by observation of colonoscopy performed by the faculty member or professor for 2 weeks. Then, they were granted to operate the sigmoidoscope for 2 weeks followed by performing colonoscopy only during the withdrawal under the supervisor’s watching in 20–30 cases. After this level of training for 2 months, they started the insertion of the scope under supervisor’s attendance. All of trainees filled the self assessment form which included patients demographics, histories, indications, and variables related to procedure, complication and pathologic report. Every 50 procedures, all trainees took feed-backs from the senior doctor for their technical assessment; cecal intubation rate, times for cecal intubation and withdrawal, complication rate, and cognitive skills including polyp and adenoma detection rate [13]. Trainees were given 15 minutes to intubate the cecum without the assistance of senior fellows or staff. SCI was defined as the successful photo-documentation of the cecal strap folds and IC valve within 15 minutes, without any assistance. We defined 15 minutes as a competent cecal intubation time based on the previous study results [14]. The nurses recorded cecal intubation time and withdrawal time in order to calculate averages. Withdrawal time did not include time spent on procedures such as biopsy and polypectomy.

### Data collection

We recorded various parameters potentially related to SCI such as age, sex, body mass index (BMI), bowel preparation scale, indication for colonoscopy, and prior surgical history. Three levels of BMI were defined as follows: thin (BMI < 18.5), normal (BMI: 18.5-24.9), over weight (BMI ≥ 25) [15]. These parameters have been analyzed at the first session of 250 cases when trainees did not reach the steady learning curve. Then next analysis was performed when they reached the steady learning curve with achieving over 90% SCI at next 250–300 cases respectively.

### Statistical analysis

All analysis was conducted using SPSS, version 18.0 (SPSS Inc., Chicago, IL, USA). The learning curve of trainees was calculated in consecutive blocks of 50 procedures. An independent t-test was used to compare the mean value. The *Chi*-square test and multivariate logistic regression analysis was used to evaluate the effect of each factor on the failure of cecal intubation. A p-value < 0.05 was considered significant.

## Results

A total of 2,050 colonoscopies performed from March 2010 to February 2012 were included in the study. Mean patient age was 57.3 ± 13.8 years, and the mean BMI was 23.9 ± 3.6. The patients included 1,236 men (60.3%) and 814 women (39.7%). Of the patients, 82.0% had no history of abdominal surgery, 5.4% had a history gynecologic surgery, and 3.1% had a previous stomach operation (subtotal gastrectomy, total gastrectomy, or partial gastrectomy). Excellence in bowel preparation was achieved in 17.7% of the patients, and good, fair and poor were 70.3%, 9.9% and 2.1%, respectively. The most common indication for colonoscopy was screening and surveillance (57.8%). During the procedure, there was 1 case of colon perforation and 5 cases of post-polyepectomy or biopsy-induced bleeding which required hospitalization. Baseline characteristics of patients and procedures before and after colonoscopic competency are summarized in Table 1. In the analyses comparing pre- and post-competency period, also, there were no significant differences in the patients factors responsible for failure except gender; Women were more prevalent in pre-competency period.

**Table 1:** Baseline characteristics of patients and procedures before and after colonoscopic competency

	**Total**	**Pre-competency**	**Post-competency**	***P *value**
Age (%)				0.447
<60	55.9	55.0	56.7	
>60	44.1	45.0	43.3	
Sex (%)				0.004
Male	60.3	57.1	63.3	
Female	39.7	42.9	36.7	
BMI (%)				0.105
18.5-25	52.1	61.7	59.1	
>25	29.0	31.4	35.5	
<18.5	18.5	6.9	5.4	
Bowel preparation (%)				0.154
Excellent/Good	88.0	86.5	91.0	
Fair/Poor	12.0	13.5	9.0	
Previous surgery (%)				
Stomach Yes	3.1	3.5	2.7	0.307
No	96.9	96.5	97.3	
Gynecologic Yes	5.4	5.7	5.0	0.557
No	94.6	94.3	95.0	
Hepatobiliary Yes	1.1	1.1	1.0	0.908
No	98.9	98.9	99.0	
Indication for colonoscopy (%)				
Screening/Surveillance	57.8	56.9	58.8	0.470
Symptomatic	34.4	34.9	33.2	0.427
Diagnostic	3.4	3.5	3.3	0.835

### Learning curve

**Figure 1:**
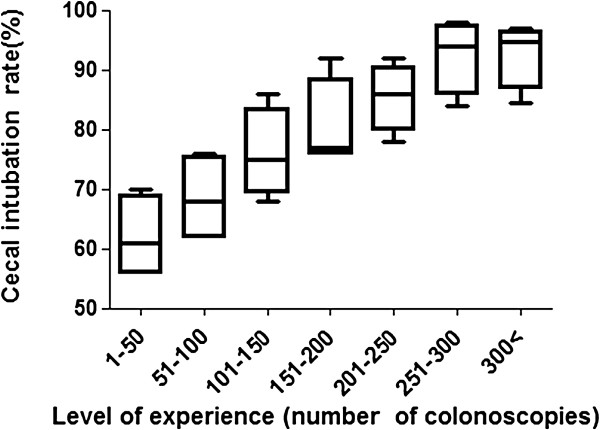
**Cecal intubation rate learning curves.** The learning curve for average successful cecal intubation rates within 15 minutes based on the number of colonoscopies is shown. (*P <* 0.05 with the Turkey test*, e*rror bars represent the 95% confidence interval). Cecal intubation rates reach the 92.5% at 250-300th procedures.

The overall success rate for successful cecal intubation in less than 15 minutes was 83.9% (1,720/2,050). The trainees’ learning curve was calculated in consecutive blocks of 50 colonoscopies. Success rates gradually increased as trainees acquired more colonoscopy experience, from 62% in the first 50 cases to 93% in the 250-300th cases (62.0%, 68.5%, 76%, 80.5%, 85.5%, 92.5% and 93.4%, respectively for every 50 consecutive blocks (Figure 1). After 250 colonoscopies, the success rate in reaching the cecum was consistently above 90% in all four fellows.

### Cecal intubation time and withdrawal time

**Figure 2:**
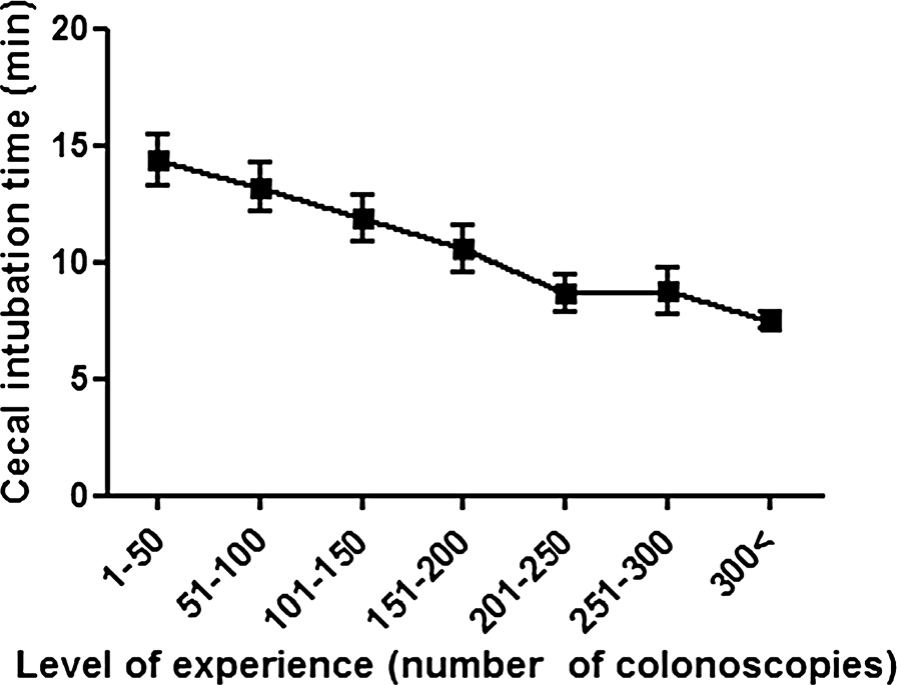
**Cecal intubation time learning curves.** The learning curve for average cecal intubation times is shown. (Error bars represent the 95% confidence interval). A significant inverse correlation between cecal intubation times and level of experience is shown.

**Figure 3:**
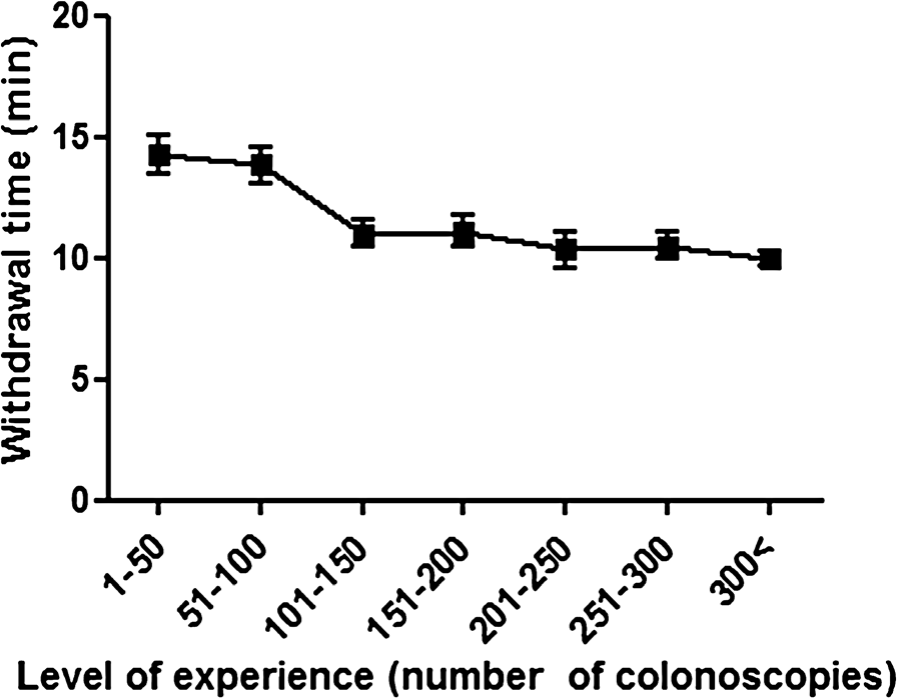
**Withdrawal time learning curves.** The learning curve for average withdrawal times is shown. (Error bars represent the 95% confidence interval). Withdrawal times decrease with the level of experience, but steady around 10 minutes after 150 procedures.

The mean cecal intubation time was 9.8 ± 6.8 minutes, and the mean withdrawal time was 11.1 ± 4.9 minutes. The mean cecal intubation time was inversely proportional to the number of colonoscopies the trainee had performed (14.4 min, 12.0 min, 8.7 min, 6.6 min for 50, 150, 250, 500 cases respectively (Figure 2). The mean withdrawal time also decreased as trainee experience increased, but plateaued around 10 minutes after 150 procedures (14.3 min, 11.1 min, 10.5 min, 9.6 min for 50, 150, 250, 500 cases respectively (Figure 3).

### Unsuccessful cecal intubation

Of the 2,050 colonoscopies, cecal intubation failed in 330 cases (16.1%). The most common anatomic site reached by the trainees during failed colonoscopies was the hepatic flexure (31.5%), followed by the transverse colon (17.9%), and sigmoid-descending junction (13.9%). In failed cases, reinsertion by the senior fellows or staff achieved SCI 97.3% of the time.

The logistic regression results are shown in Table 2. Trainees were 1.3 times as likely to fail to intubate the cecum in older patients (> 60). However, this finding was not statistically significant (p = 0.064). Trainees were 1.5 times more likely to fail to intubate the cecum in females than in males. Patients with low BMI (< 18.5) were 1.8 times more difficult to achieve SCI in than normal BMI (18.5-24.9). Additionally, patients with inadequate bowel preparation or with a history of stomach operation were more difficult for the trainees to achieve SCI in than subjects with good bowel preparation and no history of stomach operation by 2.1 times and 3.3 times, respectively. The Hosmer-Lemeshow goodness-of-fit test indicated that this model was a good fit for the data (p = 0.855). A significant difference in the cecal intubation rate was not found according to the history of gynecologic and hepatobiliary surgery, indication for colonoscopy, or ASA status.

**Table 2:** Factors affecting the cecal intubation failure by logistic regression analysis

	**Failure rate (%)**	**Odds ratio**	**95% CI**	***P *value**
Age				
<60	13.6	1		
>60	19.3	1.287	0.985-1.681	0.064
Sex				
Male	13.7	1		
Female	19.8	1.518	1.164-1.980	0.002
BMI				
18.5-25	15.7	1		
>25	12.6	0.808	0.600-1.088	0.159
<18.5	26.9	1.771	1.108-2.830	0.017
Bowel preparation				
Excellent/Good	14.8	1		
Fair/Poor	25.6	2.072	1.464-2.933	0.000
Previous surgery				
Stomach No	15.3	1		
Yes	41.3	3.323	1.892-5.835	0.000
Gynecologic No	15.7	1		
Yes	23.6	1.422	0.820-2.412	0.201

After 250 procedures, the success rate for cecal intubation stabilized at over 90%. Because of this, we compared the predictive factors before and after 250 procedures. Interestingly, by logistic regression analysis, age and gender did not show any difference in cecal intubation rate in the trainees before achieving competence. Only low BMI, inadequate bowel preparation, and previous stomach surgery were associated with cecal intubation failure: 1.9 times, 2.0 times, and 2.2 times more likely, respectively. After trainees achieved competency, poor bowel preparation and history of stomach surgery were associated with the failure of cecal intubation. The results are summarized in Table 3.

**Table 3:** Factors affecting the cecal intubation failure before and after colonoscopic competency by logistic regression analysis

	**Pre-competency**				**Post-competency**			
	**Failure rate (%)**	**Odds ratio**	**95% CI**	***p-*value**	**Failure rate (%)**	**Odds ratio**	**95% CI**	***p-*value**
Age								
<60	22.0	1			5.7	1		
>60	29.8	1.210	0.872-1.678	0.253	9.0	1.310	0.773-2.219	0.316
Sex								
Male	22.4	1			6.2	1		
Female	29.6	1.325	0.953-1.842	0.095	8.8	1.668	0.973-2.859	0.063
BMI								
18.5-24.99	25.8	1			6.6	1		
≥25	21.3	0.783	0.545-1.124	0.185	5.9	1.008	0.565-1.798	0.978
< 18.5	40.4	1.902	0.986-3.189	0.028	11.8	1.370	0.521-3.607	0.524
Bowel preparation								
Excellent/Good	23.5	1			6.5	1		
Fair/ Poor	39.7	2.037	1.312-3.162	0.002	12.0	2.370	1.240-4.529	0.008
Previous surgery								
Stomach No	24.6	1			6.6	1		
Yes	51.4	2.189	1.044-4.587	0.002	28.6	6.643	2.651-16.650	0.000
Gynecologic No	24.8	1			7.0	1		
Yes	36.8	1.609	0.838-3.088	0.153	9.4	0.931	0.272-3.183	0.909

## Discussion

To perform a colonoscopy safely and effectively, the ability to reach and examine the cecum is necessary. Thus, successful cecal intubation is an obligatory measure of competence. Previous reports indicate that experienced endoscopists consistently achieve cecal intubation in more than 90% of their procedures, and rates above 90% have generally been the benchmark for competency [16,17]. However, cecal intubation rates in the early phase of training are nowhere near this benchmark [3-6]. Our study considered cecal intubation successful only if cecal landmarks (cecal strap fold and ileocecal valve) were clearly photo-documented within 15 minutes without assistance. The success rate in trainees’ fist 50 procedures was only 62%, which is in agreement with previous results [4,5]. The success rate for cecal intubation was 62.0%, 68.5%, 76.0%, 80.5%, 85.5%, 92.5% and 93.4% for every 50 consecutive blocks, respectively. After 250 procedures, the success rate steadily increased above 90%. Thus, in our study we assessed the colonoscopic competence based on the first 250 procedures, and attempted to discern predictors of SCI before and after trainees achieved competency.

Prolonged cecal intubation can cause patients discomfort, barotraumas, or consecutive reinsertion failure, even at the hand of an experienced colonoscopist. Thus, it is important to determine and to avoid factors that affect cecal intubation failure by trainees, especially in the early learning phase. The results in this study showed that predictive factors for cecal intubation failure by trainees were female gender, low BMI, poor bowel preparation, and prior stomach operation. Women had a lower cecal intubation rate than men, which was also reported in previous studies [4,5]. This could be explained by the fact that the colon is typically longer in women, women may have more unknown anatomic variations, or because of complications as a result of previous gynecologic surgeries [18,19]. However, gynecologic surgery did not show any significance for cecal intubation failure in this study.

In patients with low BMI (<18.5), trainees were more likely to fail to reach the cecum within 15 minutes, which is in agreement with results from in a previous study [4]. Patients with a low BMI had a sigmoid colon that was more redundant or difficult [10]. One possible explanation for this finding may be lower amounts of fat, which provides resistance to the colonoscope and thus helps to decrease sigmoid mobility.

There have been no previous reports regarding the relationship between colonoscopy and stomach surgery. In subtotal or total gastrectomy, the surgeon dissects the greater omentum, which is attached to stomach and transverse colon. This may result in the formation of adhesions and anatomic variations around the transverse colon [20]. A significant difference in the cecal intubation failure rate was associated with patient history of stomach operation in the study. In contrast with gynecologic or hepatobiliary operation, trainees had only a 58.7% success rate for cecal intubation if the patient had a history of stomach operation. In the cases of stomach operation, the most common anatomic site reached by the trainees during failed colonoscopies was the transverse colon (38.5%) and sigmoid-descending junction (34.6%). In addition, most trainees in the early phase of colonoscopy insert the colonoscope by push-type, and try to reduce the loop after reaching the transverse colon. However, if there are adhesions around the transverse colon, there may be insufficient shortening of the colon, thus creating difficulties in the straightening of the scope.

In this study, 250 procedures were required for trainees to achieve competence in colonoscopy within 15 minutes. These results are very similar to those of previous studies [6,14]. This supports a previous study that an average of 275 procedures performed within 16 minutes are needed for the average GI trainee to meet minimal competence criteria. Two prior studies showed that the success rate significantly improved and trainees reached the requisite standard competence (>90%) after 150–200 cases, which is even less than the 250 cases determined in our study [4,5]. The reason for this discrepancy is probably because our study gave trainees less time to intubate the cecum. The published benchmarks for cecal insertion time for minimal competency range from 15 to 20 minutes [6,14]. Even in the first 50 cases, the mean time to reach the cecum was less than 15 minutes and decreased as trainees acquired more experience.

There are several limitations to our study. First, it may be difficult to generalize these predictive factors for cecal intubation failure since this is a single-center study. Also, we considered only the patient’s factors, and did not look at factors such as assistants’ experience, or the fatigue of the colonoscopist. In addition, it is clear that fellows acquired the skill at different rates, but we did not consider the differences between fellows by this measure. The number of trainees participated and the number of subjects with previous stomach surgery included in this study was relatively small. Furthermore, the study patients may pose a selected group because the majority of procedures were indicated by screening or surveillance. For ethical reasons, however, we included only patients with good general condition (ASA class 1 or 2) in order to avoid potential risks of procedure related complications from the trainee’s incomplete skill acquaintance. Finally, colonoscopy method using assistant device or technique such as cap or water immersion was not taught to the trainees when they failed the cecal intubation. Several kinds of technical assistances may be helpful for trainees to perform difficult colonoscopy. In particular, there are emerging data that warm water immersion (WWI) technique enhanced cecal intubation rate and willingness to undergo a repeat colonoscopy. Also, WWI can not only reduce the number of patients requiring on-demand sedation but also improve the overall patients tolerance of colonoscopy. Therefore, it would be very interesting whether this technique facilitates the learning curve in the trainee [21,22].

## Conclusion

Several patient characteristics were identified that may predict difficulty of cecal intubation in colonoscopy trainees. The results could be informative so that trainees enhance the success rate regarding better colonoscopy training programs, as well as that they ensure patient safety in the early phase of the learning curve. Particularly, before trainees acquire competence, it might be advised for trainees to avoid performing this procedure on patients who are of low BMI, have inadequate bowel cleansing, or a history of stomach surgery.

## Competing interests

The authors declare that they have no competing interests.

## Authors’ contributions

This study was supported by a grant of the Korean Health Technology R&D Project, Ministry for Health, Welfare & Family Affairs (A102065-23) and a grant from the National R&D Program for Cancer Control, Ministry of Health & Welfare, Republic of Korea (1220230). All authors read and approved the final manuscript.

## Pre-publication history

The pre-publication history for this paper can be accessed here:

http://www.biomedcentral.com/1472-6920/13/5/prepub
